# Cannibalism prevents evolutionary suicide of ontogenetic omnivores in life‐history intraguild predation systems

**DOI:** 10.1002/ece3.5004

**Published:** 2019-02-27

**Authors:** Vincent Hin, André M. de Roos

**Affiliations:** ^1^ Institute for Biodiversity and Ecosystem Dynamics University of Amsterdam Amsterdam The Netherlands

**Keywords:** cannibalism, eco‐evolutionary dynamics, life‐history intraguild predation, omnivory, stage structure

## Abstract

The majority of animal species are ontogenetic omnivores, that is, individuals of these species change or expand their diet during life. If small ontogenetic omnivores compete for a shared resource with their future prey, ecological persistence of ontogenetic omnivores can be hindered, although predation by large omnivores facilitates persistence. The coupling of developmental processes between different life stages might lead to a trade‐off between competition early in life and predation later in life, especially for ontogenetic omnivores that lack metamorphosis. By using bioenergetic modeling, we study how such an ontogenetic trade‐off affects ecological and evolutionary dynamics of ontogenetic omnivores. We find that selection toward increasing specialization of one life stage leads to evolutionary suicide of noncannibalistic ontogenetic omnivores, because it leads to a shift toward an alternative community state. Ontogenetic omnivores fail to re‐invade this new state due to the maladaptiveness of the other life stage. Cannibalism stabilizes selection on the ontogenetic trade‐off, prevents evolutionary suicide of ontogenetic omnivores, and promotes coexistence of omnivores with their prey. We outline how ecological and evolutionary persistence of ontogenetic omnivores depends on the type of diet change, cannibalism, and competitive hierarchy between omnivores and their prey.

## 1 INTRODUCTION

Ontogenetic (or life‐history) omnivores are species that change or expand their diet during life (Persson, 1988; Pimm & Rice, 1987). These include organisms with direct development that change resources as a result of body size growth (such as many fish species), as well as species with indirect development, in which the change in diet is associated with a metamorphosis (e.g., holometabolous insects). The majority of all animal species fall within these two categories, with the exception of most birds and mammals (Werner, 1988; Werner & Gilliam, 1984; Wilbur, 1980). The omnipresence of ontogenetic omnivores within the animal kingdom certainly suggests a high evolutionary potential of such a strategy.

The apparent evolutionary success of ontogenetic omnivores seems to be at odds with the insight that ontogenetic omnivores without a metamorphosis can suffer from a trade‐off that limits the ability to specialize on different resources (Hjelm, Persson, & Christensen, 2000; Hjelm, van de Weerd, & Sibbing, 2003; Robinson, Wilson, & Shea, 1996; Schluter, 1995). Resource specialization means that body morphology, physiology, and behavior (among others) are optimally adjusted to search, capture, and process a specific resource. Because different resources often require a different set of optimal traits, specialist species are better adapted to utilize a specific resource than generalists. Ontogenetic omnivores that lack metamorphosis cannot rearrange their body morphology and physiology along with the change in resource use, and this limits ecological specialization on resources used in different life stages (Ebenman, 1992; Moran, 1994; Werner, 1988; Werner & Gilliam, 1984). Such an ontogenetic trade‐off becomes especially important if the resources used in different life stages are increasingly distinct in terms of the morphology that is required to adequately handle them (Hjelm et al., 2000, 2003). Because of the ontogenetic trade‐off in resource specialization, ontogenetic omnivores are considered less efficient consumers than their specialist competitors (Byström, Ask, Andersson, & Persson, 2013; Persson, 1988). This makes ecological persistence of omnivores vulnerable to competition with specialist consumer species (Hin, Schellekens, Persson, & de Roos, 2011; Toscano, Hin, & Rudolf, 2017). In fact, metamorphosis is hypothesized to have evolved as a mechanism to decouple developmental processes between different life stages, such that each life stage can specialize independently and escape the negative effects of competition (Ebenman, 1992; Moran, 1994; Ten Brink & de Roos, 2017; Werner, 1988).

**Figure 1:**
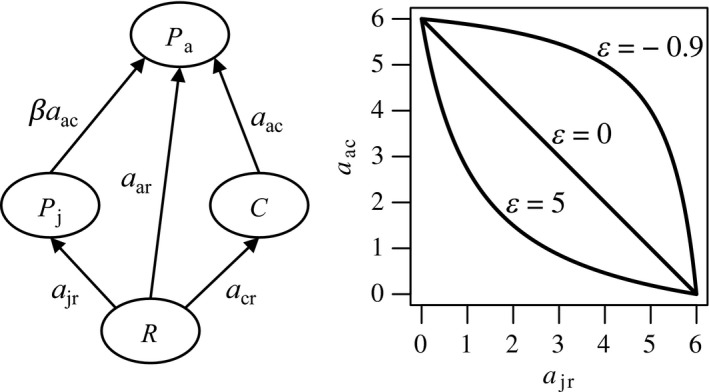
Left: The life‐history intraguild predation food web. Cannibalistic adult intraguild predators (*P*_a_) feed on juvenile intraguild predators (*P*_j_) and consumers (*C*) with attack rate parameter *a*_ac_. Cannibalistic preference is regulated by parameter *β*. Resource feeding by adult intraguild predators, juvenile intraguild predators, and consumers is regulated by attack rate parameters *a*_ar_, *a*_jr_ and *a*_cr_, respectively. Juvenile and adult intraguild predators are furthermore connected through the life‐history processes of maturation and reproduction (links not shown). Right: the ontogenetic trade‐off function (Equation (1)) between attack rate parameters *a*_jr_ and *a*_ac_ depending on the shape parameter *ε*. We distinguish between a weak trade‐off (*ε* = −0.9), a linear trade‐off (*ε* = 0), and a strong trade‐off (*ε* = 5)

Ecological persistence of ontogenetic omnivores has mainly been studied in the context of life‐history intraguild predation (LHIGP; Pimm & Rice, 1987; Polis, Myers, & Holt, 1989), in which small/juvenile ontogenetic omnivores (intraguild predators) compete with a specialist consumer (intraguild prey) for a shared resource, while large/adult omnivores prey on the consumer (Figure 1). Toscano et al. (2017) furthermore distinguish diet broadening LHIGP, in which adult omnivores also feed on the resource, from diet shift LHIGP, in which they do not. Both scenarios give rise to alternative stable states, with one state in which predators persist and one in which they are absent. The state without predators is stable because consumers suppress the resource to a level that is insufficient for growth and successful maturation of juvenile predators (Hin et al., 2011; Persson & Greenberg, 1990; Van de Wolfshaar, De Roos, & Persson, 2006). In the state with predators, adult intraguild predators either top‐down control (diet shift LHIGP) or exclude consumers (diet broadening LHIGP). This increases resource availability and allows for growth and maturation of juvenile intraguild predators (cultivation hypothesis; Walters & Kitchell, 2001).

In addition, adult intraguild predators are often cannibalistic and feed on their juvenile conspecifics (Byström et al., 2013). Cannibalism reduces the ability of the predator to top‐down control its prey, and the consequences of this effect depend on the competitive hierarchy between predators and consumers. If consumers are the superior resource competitors, cannibalism is detrimental for predator persistence, because it releases top‐down control of consumers and breaks down the cultivation effect (Toscano et al., 2017). Instead, if (juvenile) predators are the superior resource competitors, cannibalism promotes coexistence with prey, because it reduces top‐down control on the resource, which allows the consumers to persist (Toscano et al., 2017). Conclusively, ecological persistence of ontogenetic omnivores in LHIGP systems depends on (a) the competitive hierarchy between (juvenile) predators and consumers, (b) the ability of top‐down control by adult predators, and (c) the level of cannibalism.

All three interactions are subject to the ontogenetic trade‐off in resource specialization, but currently there is little insight how this trade‐off will affect persistence of intraguild predators and coexistence with prey, nor how resource specialization will evolve within (cannibalistic) LHIGP systems. Using a quantitative genetics approach within a Lotka–Volterra model, Patel and Schreiber (2015) studied eco‐evolutionary dynamics in an unstructured intraguild predation (IGP) community, in which the intraguild predator experiences a trade‐off between feeding on the resource versus feeding on the prey. Depending on the strength of this trade‐off, a number of outcomes were possible, such as stabilizing selection toward a generalist or specialist phenotype, eco‐evolutionary cycles between the two specialized phenotypes, and evolutionary suicide of the intraguild predator (Patel & Schreiber, 2015). Evolutionary suicide occurs if a population adapts toward self‐extinction by crossing a discontinuous change in the model's equilibrium structure (e.g., a fold bifurcation; Parvinen, 2005; Ferrière & Legendre, 2013; Parvinen, 2016). Given the prevalence of alternative stable states in LHIGP systems, it seems likely that evolutionary suicide also occurs in size‐structured IGP systems that result from ontogenetic diet changes of the intraguild predator.

Here, we use a stage‐structured bioenergetics modeling approach to study the conditions for ecological and evolutionary persistence of ontogenetic omnivores within life‐history intraguild predation systems. Building on the models used by Hin et al. (2011) and Toscano et al. (2017), we study ecological equilibrium patterns and evolutionary dynamics of resource specialization of intraguild predators depending on the level of cannibalism and the type of diet change during predator ontogeny (diet shift or diet broadening). We assume that intraguild predators are subject to an ontogenetic trade‐off in resource specialization. This trade‐off entails that increased specialization of juvenile intraguild predators on the resource comes at the cost of decreased specialization of adult intraguild predators toward intra‐ and interspecific predation, and vice versa. Consequently, we assume that intraguild predators lack any form of metamorphosis and that predation by adult predators requires a different functional morphology than resource feeding by juvenile intraguild predators. We find that noncannibalistic intraguild predators evolve toward an abrupt threshold in the ecological dynamics beyond which they go extinct. This evolutionary suicide of intraguild predators occurs irrespective of the type of diet change. Cannibalism stabilizes evolutionary dynamics and can prevent evolutionary suicide. We assess model robustness with respect to maximum resource density and trade‐off shape. We conclude that the evolutionary success of nonmetamorphosing ontogenetic omnivores in LHIGP systems can be explained by cannibalism and resource specialization of juveniles.

## 2 MODEL AND METHODS

### 2.1 Model formulation

We build upon the LHIGP model of Hin et al. (2011) and Toscano et al. (2017). Both use the stage‐structured bioenergetics modeling approach as described by De Roos et al. (2007, 2008), which extends the bioenergetic model presented by Yodzis and Innes (1992) by separating total biomass into juvenile and adult stages. Stage‐structured is considered for the intraguild predator by distinguishing adult *P*_a_ and juvenile *P*_j_ predator biomass. The intraguild predator exhibits a diet shift/broadening during ontogeny and is potentially cannibalistic (Figure 1), such that adult predators feed on juvenile conspecifics. Because stage‐specific interactions are assumed to be absent for the resource (*R*) and the consumer (*C*), we do not have to account for stage structure in these species. Although stage‐specific interactions for the consumer can potentially yield novel and interesting results, such an extension falls outside the scope of the current study.

Functional responses of the consumer and the intraguild predator are formulated in terms of an attack rate (area searched for prey per unit time) and a handling time constant. Following Ten Brink and de Roos (2017), we use the attack rate parameter to perform evolutionary analysis of resource specialization. Within each life stage, attack rates are assumed to scale linearly with individual body size with proportionality constant *a*_*ik*_ for species/stage *i* feeding on species/stage *k*, where *i* and *k* can equal r, c, j, or a, corresponding to resource, consumer, juvenile, and adult predators, respectively. In the following, we use “attack rate” to refer to this proportionality constant. Consumers and juvenile predators feed solely on the resource with attack rates *a*_cr_ and *a*_jr_, respectively. Adult predators search for resource and prey (both consumer and juvenile predators) with attack rates *a*_ar_ and *a*_ac_, respectively. The ontogenetic trade‐off in resource specialization is implemented between the juvenile attack rate on the resource (*a*_jr_) and the adult attack rate (*a*_ac_) for predation as follows (modified from Ebenman, 1992):

(1) $$a_{\mathrm{ac}}=\frac{a_{p}-a_{\mathrm{jr}}}{1+\varepsilon a_{\mathrm{jr}}/a_{p}}$$

Consequently, adaptation to interspecific predation is assumed to also increase rates of cannibalism. In Equation (1), *a*_p_ is the maximum value that *a*_jr_ and *a*_ac_ can adopt, while *ε* controls the shape of the trade‐off. For *ε* = 0, the trade‐off is linear, while for −1 < *ε* < 0 the trade‐off is weak and concave from below, and for *ε* > 0, the trade‐off is strong and convex from below (Figure 1). The adult attack rate for resource feeding (*a*_ar_) is not part of Equation (1) and determines the different diet change scenarios (diet shift: *a*_ar_ = 0 or diet broadening; *a*_ar_ > 0), as well as the competitive hierarchy between consumers and intraguild predators (see Section 2.3 and Appendix).

Furthermore, we follow Toscano et al. (2017) and adopt the parameter *β* to control cannibalistic preference. Cannibalistic preference *β* scales the rate of cannibalistic feeding relative to the predation attack rate *a*_ac_ (Table 1 and Figure 1). Handling time is represented by the proportionality constant *h*_*i*_, relating handling time to the inverse of individual body mass of species *i*. This parameter is assumed equal across all prey types. The mass‐specific biomass ingestion rate of adult predators (*P*_a_) feeding on juvenile predators (*P*_j_), consumers (*C*), and the resource (*R*) is given by:

(2) $$I_{a}=\frac{a_{\mathrm{ar}}R+a_{\mathrm{ac}}\left(C+\beta P_{j}\right)}{1+h_{p}\left(a_{\mathrm{ar}}R+a_{\mathrm{ac}}\left(C+\beta P_{j}\right)\right)}$$

**Table 1:** Model equations

Description	Equation
Pred. attack rate trade‐off	$a_{\mathrm{ac}}=\frac{a_{p}-a_{\mathrm{jr}}}{1+\varepsilon a_{\mathrm{jr}}/a_{p}}$
Consumer ingestion^a^	$I_{c}=\frac{a_{\mathrm{cr}}R}{1+h_{c}a_{\mathrm{cr}}R}$
Juvenile pred. ingestion^a^	$I_{j}=\frac{a_{\mathrm{jr}}R}{1+h_{p}a_{\mathrm{jr}}R}$
Adult pred. ingestion^a^	$I_{a}=\frac{a_{\mathrm{ar}}R+a_{\mathrm{ac}}\left(C+\beta P_{j}\right)}{1+h_{p}\left(a_{\mathrm{ar}}R+a_{\mathrm{ac}}\left(C+\beta P_{j}\right)\right)}$
Consumer net biomass production^a^	$\nu_{c}=\sigma I_{c}-T_{c}$
Juvenile pred. net biomass production^a^	$\nu_{j}=\sigma I_{j}-T_{p}$
Adult pred. net biomass production^a^	$\nu_{a}=\sigma I_{a}-T_{p}$
Juvenile pred. mortality rate	$D_{j}=\mu_{p}+\frac{a_{\mathrm{ac}}\beta P_{a}}{1+h_{p}\left(a_{\mathrm{ar}}R+a_{\mathrm{ac}}\left(C+\beta P_{j}\right)\right)}$
Juvenile pred. maturation rate^a^	$\gamma \left(\nu_{j},D_{j}\right)=\frac{\nu_{j}-D_{j}}{1-z^{1-D_{j}/\nu_{j}}}$
Resource biomass dynamics	$\frac{dR}{dt}=\delta \left(R_{\max}-R\right)-I_{c}C-I_{j}P_{j}-\frac{a_{\mathrm{ar}}R}{1+h_{p}\left(a_{\mathrm{ar}}R+a_{\mathrm{ac}}\left(C+\beta P_{j}\right)\right)}P_{a}$
Consumer biomass dynamics	$\frac{dC}{dt}=\nu_{c}C-\frac{a_{\mathrm{ac}}P_{a}}{1+h_{p}\left(a_{\mathrm{ar}}R+a_{\mathrm{ac}}\left(C+\beta P_{j}\right)\right)}C-\mu_{c}C$
Juvenile pred. biomass dynamics	$\frac{dP_{j}}{dt}=\nu_{a}P_{a}+\nu_{j}P_{j}-\gamma \left(\nu_{j},D_{j}\right)P_{j}-D_{j}P_{j}$
Adult pred. biomass dynamics	$\frac{dP_{a}}{dt}=\gamma \left(\nu_{j},D_{j}\right)P_{j}-\mu_{p}P_{a}$

a Mass‐specific rate.

Functional responses of consumers and juvenile predators feeding on the resource follow from identical considerations, with the appropriate parameters (see Table 1).

For adult predators, *I*_a_ is converted to mass‐specific net biomass production ($\nu_{a}$) by multiplication with conversion efficiency *σ* and subtraction of mass‐specific maintenance rate *T*_a_ (Table 1). Mass‐specific net biomass production of consumers ($\nu_{c}$) and juvenile predators ($\nu_{j}$) relates in a similar vein to ingestion (Table 1). Mass‐specific net biomass production rates determine rates of growth and reproduction, and therefore, a species can only have a positive equilibrium density if their mass‐specific net biomass production rate is positive (De Roos et al., 2007).

Dynamics of resource, consumer, juvenile, and adult predator biomass are described by four ordinary differential equations (ODEs: Table 1). Resource biomass increases following semichemostat dynamics (De Roos & Persson, 2013) with turnover rate *δ* and maximum resource density *R*_max_ and decreases due to ingestion by consumers and predators. Consumer biomass increases with total consumer net biomass production, $\nu_{c}C$ and decreases through total feeding by adult predators and consumer background mortality $\mu_{c}C$. Juvenile biomass decreases due to background mortality with rate *μ*_p_ and through cannibalism by adult predators. Juvenile mortality due to cannibalism amounts to:

(3) $$\frac{a_{\mathrm{ac}}\beta P_{a}}{1+h_{p}\left(a_{\mathrm{ar}}R+a_{\mathrm{ac}}\left(C+\beta P_{j}\right)\right)}$$

Juvenile biomass increases through reproduction, which depends on the total net biomass production of adult predators ($\nu_{a}P_{a}$). Juvenile predators use their net biomass production ($\nu_{j}P_{j}$) exclusively for somatic growth. Growth increases juvenile biomass and, when positive, ultimately leads to maturation of juveniles to the adult stage. The maturation rate $\gamma (\nu_{j},D_{j})$ depends on mass‐specific net biomass production $\nu_{j}$, juvenile mortality, and the ratio between size at birth and size at maturation (*z*). The maturation function (see Table 1) is derived such that the stage‐structured biomass model in equilibrium is identical to a model with a continuous size structure (De Roos et al., 2008). Therefore, we implicitly account for the continuous population size‐structure dynamics of the predator (see De Roos et al., 2008, for details). Adult biomass decreases through background mortality with rate *μ*_p_ and only increases through maturation as adult predators spend all net biomass production on reproduction (Table 1). Previous studies have shown that this stage‐structured bioenergetics approach yields results that are in good qualitative agreement with a continuously size‐structured model, in which the adult predator life stage exhibits indeterminate growth in body size (De Roos & Persson, 2013; Ten Brink & de Roos, 2017).

### 2.2 Model parameters

Consumer model parameters are taken from Hin et al. (2011). This parameterization is based on the empirical relationships that across different species the mass‐specific rates of maximum ingestion (which equals the inverse of handling time), maintenance, and mortality scale with adult body size to the power −0.25 (see also De Roos & Persson, 2013). Consumer parameters for handling time, mass‐specific maintenance, and mortality are, respectively, *h*_c_ = 0.1, *T*_c_ = 1, and *μ*_c_ = 0.1. Hin et al. (2011) furthermore adopt a half‐saturation constant of 1, and since the half‐saturation constant equals the ratio between maximum ingestion and attack rate, this leads to a consumer attack rate value of *a*_cr_ = 10.

For the predator parameters, we deviate from Hin et al. (2011) and instead use the finding of Brose et al. (2006) that the geometric average predator–prey body mass ratio is 42. Combining this ratio with the empirical relationships described above leads to predator parameter values of *h*_p_ = 0.25, *T*_p_ = 0.4, and *μ*_p_ = 0.04. Maximum resource density (*R*_max_), predator attack rate parameters, and the cannibalistic constant *β* are varied upon analysis, but the maximum attack rate used in Equation (1) is set to *a*_p_ = 6. This equals 1.5 times the default value that results from using a half‐saturation constant of 1. Furthermore, we follow Hin et al. (2011) and Toscano et al. (2017) by adopting σ=0.5, δ=1, and *z* = 0.01. The latter value implies that consumer body size overlaps with the size range of juvenile predators, which seems appropriate for competing species (Cohen, Pimm, Yodzis, & Saldaña, 1993; Woodward & Hildrew, 2002).

### 2.3 Model analysis

We used PSPManalysis (De Roos, 2018) to study equilibrium and evolutionary dynamics. PSPManalysis is a software package with numerical procedures to perform demographic, bifurcation, and evolutionary analysis of physiologically structured population models. In addition, we used MatCont (Dhooge, Govaerts, & Kuznetsov, 2003), a MATLAB package for numerical bifurcation analysis to assess equilibrium stability. An equilibrium is (locally) stable against invasion or extinction of a certain species if all eigenvalues of the Jacobian matrix evaluated at that particular equilibrium have negative real parts. Limit cycles only occur in a small region of parameter space and their amplitude is insignificant, for which reason we will not consider them further.

PSPManalysis uses adaptive dynamics as the framework for evolutionary analysis (Dieckmann & Law, 1996; Geritz, Kisdi, Meszéna, & Metz, 1998; Metz, Geritz, Meszéna, Jacobs, & Van Heerwaarden, 1996). In adaptive dynamics, evolutionary change occurs through mutant phenotypes (*y*′) that invade and take over the population dynamical attractor of the resident phenotype (*y*). Invasion and replacement can only be successful for mutants with phenotypes in the direction of the selection gradient. The selection gradient is sign equivalent with the derivative of the mutant's lifetime reproductive success, $R_{0}\left(y,y^{\prime }\right)$, with respect to the mutant's phenotype and evaluated at $y^{\prime }=y:\left(\partial R_{0}\left(y^{\prime },y\right)/\partial y^{\prime }{|}_{y^{\prime }=y}\right)$ (Geritz et al., 1998). Evolutionary change stops when the selection gradient becomes zero. Such an evolutionary singular strategy (ESS) has two different stability properties. Convergence stability tells whether gradual evolution moves toward the ESS (convergence stable) or away from the ESS (convergence unstable). Evolutionary stability refers to whether the monomorphic population can evolve into a dimorphic population (evolutionary unstable; the mutant and resident can coexist; see also Geritz et al., 1998), or not (evolutionary stable). PSPManalysis calculates the selection gradient numerically and detects and classifies ESSs according to these stability properties. We therefore assume that invading mutants with a trait value in the direction of the selection gradient will be able to successfully oust the resident phenotype. Although we do not assess this directly, Geritz, Gyllenberg, Jacobs, and Parvinen (2002) show that this assumption is readily satisfied in ecologically realistic models, as long as the resident attractor is sufficiently far from any population dynamical bifurcation.

We compare equilibrium and evolutionary properties of the model as a function of *a*_jr_ (negatively related to *a*_ac_ according to Equation (1)) between cannibalistic (*β* = 1) and noncannibalistic (*β* = 0) predator populations and for three different values of *a*_ar_ (0, 3, and 4). These three values cover the two diet change scenarios: diet shift for *a*_ar_ = 0 and diet broadening for *a*_ar_ = 3 and *a*_ar_ = 4. In addition, the value of *a*_ar_ allows for different kinds of competitive hierarchy between consumers and intraguild predators: For *a*_ar_ = 0 and *a*_ar_ = 3, consumers persist on lower resource levels and outcompete the intraguild predator irrespective of the value of *a*_jr_; for *a*_ar_ = 4, the predator becomes the superior resource competitor if *a*_jr_ > 4 (see Appendix for an overview and a derivation of these different cases). We check robustness of the results with respect to the level of cannibalism (*β*), maximum resource density (*R*_max_), and trade‐off shape (*ε*).

In this three species community, four different types of population dynamical equilibria are possible (with corresponding abbreviations): a resource‐only equilibrium (R‐equilibrium), a consumer–resource equilibrium (CR‐equilibrium), a predator–resource equilibrium (PR‐equilibrium), and a predator–consumer–resource equilibrium (PCR‐equilibrium, also called coexistence state). Except for low values of maximum resource density (*R*_max_ < 0.2820), the R‐equilibrium is unstable because it can always be invaded by consumers.

## 3 RESULTS

### 3.1 Diet shift of intraguild predators

**Figure 2:**
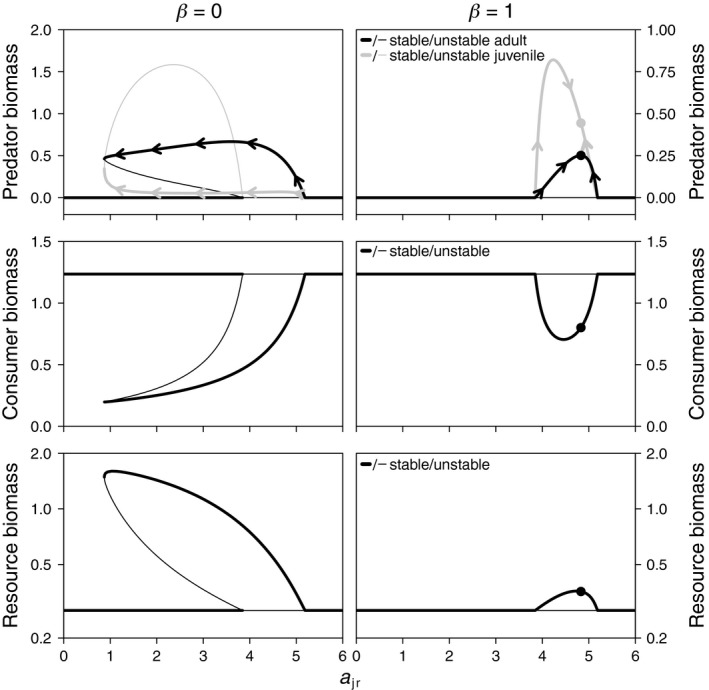
Model equilibria as a function of juvenile resource specialization (*a*_jr_; with concomitant change in *a*_ac_ following Equation (1)) for the diet shift scenario (adult predators do not feed on the resource: *a*_ar_ = 0). Evolutionary change (indicated with arrowheads in top panels) of *a*_jr_ in the stable coexistence equilibrium leads to evolutionary suicide of noncannibalistic predators (*β* = 0; left panels) and to a convergence and evolutionary stable ESS with cannibalistic predators (*β* = 1; right panels ESS is indicated with dots). Note difference in vertical axis scaling for top panels. Other parameters are *a*_cr_ = 10, *a*_p_ = 6, *ε* = 0, *h*_p_ = 0.25, *h*_c_ = 0.1, *T*_p_ = 0.4, *T*_c_ = 1.0, *μ*_p_ = 0.04, *μ*_c_ = 0.1, *z* = 0.01, *σ* = 0.5, *R*_max_ = 3, and *δ* = 1

For *a*_ar_ = 0, adult predators do not feed on the resource and require consumer biomass for reproduction (diet shift scenario). Consequently, predators can only persist in a coexistence state (Figure 2). In this coexistence state, adult predators top‐down control consumers and thereby release competition between consumers and juvenile intraguild predators (Hin et al., 2011; Toscano et al., 2017). Without cannibalism (*β* = 0, Figure 2), this coexistence state is stable for a broad range of intermediate *a*_jr_‐values (with concomitant change in *a*_ac_ following Equation (1)). For low juvenile resource specialization (*a*_jr_ < 3.55), the coexistence state occurs alternative to a stable CR‐equilibrium in which predators cannot invade. This is because the resource density in the CR‐equilibrium is insufficient for somatic growth of juvenile predators at low *a*_jr_‐values (see Appendix). Predators can invade the CR‐equilibrium if juveniles specialize on resource feeding and do not suffer from competition with consumers (high *a*_jr_). This renders the CR‐equilibrium unstable at high *a*_jr_‐values. At even higher *a*_jr_, the interspecific predation attack rate *a*_ac_ becomes too low for predator persistence.

With a diet shift and otherwise default parameters, evolution of resource specialization under an ontogenetic trade‐off results in evolutionary suicide of noncannibalistic predators through directional selection toward high adult predation attack rates (*a*_ac_). In the coexistence state, suppression of consumers by adult predators leads to low consumer biomass and high resource biomass. Consequently, competition acts most strongly in the adult life stage of the predator. This is indicated by a low juvenile‐adult biomass ratio, due to rapid juvenile growth and maturation and low reproduction rates (Figure 2). The fierce competition between adults selects for a higher predation attack rate (*a*_ac_), at the expense of lower juvenile feeding ability (*a*_jr_; Figure 2 top left panel, arrows along PCR‐equilibrium indicate direction of evolutionary change). Ultimately, increasing *a*_ac_ and decreasing *a*_jr_ drive the predator population beyond the ecological threshold (fold bifurcation) that marks the minimum level of *a*_jr_ for which predator persistence is possible. Beyond this threshold, the system will converge to the stable CR‐equilibrium, from which predator re‐invasion is impossible.

Cannibalism in the predator population with a diet shift disrupts the occurrence of a stable coexistence state as alternative to the stable CR‐equilibrium and stabilizes selection on the resource specialization trade‐off (Figure 2; right panels). The disappearance of the coexistence state at low *a*_jr_‐values occurs because cannibalism reduces top‐down control of adult predators on consumers, thereby annulling the competitive release of juvenile intraguild predators (Toscano et al., 2017). With cannibalism, predators persist only for a limited range of high *a*_jr_‐values, in which there is an ESS that is convergent and evolutionarily stable (indicated in Figure 2, right panels). Because cannibalism disrupts top‐down control of consumers by adult predators, competition acts most strongly in the juvenile stage, especially for lower *a*_jr_‐values. This leads to positive selection on *a*_jr_ below the ESS, while in the noncannibalistic case, the selection gradient for similar *a*_jr_‐values is negative. From an evolutionary point of view, cannibalism promotes predator persistence by preventing evolutionary suicide that results from selection toward increasing adult specialization.

**Figure 3:**
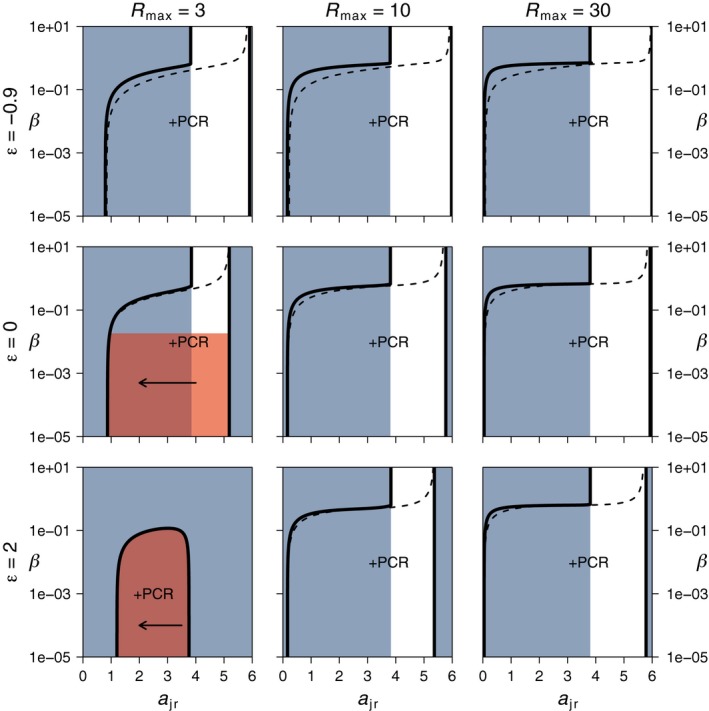
In the diet shift scenario (*a*_ar_ = 0), the occurrence of evolutionary suicide depends on maximum resource density (*R*_max_) and trade‐off shape (*ε*). For *R*_max_ = 3 and *ε* = 0, as well as *R*_max_ = 3 and *ε* = 2, evolutionary suicide (indicated with red shading) occurs at low levels of cannibalism (vertical axis). Here, there is no ESS for *a*_jr_ on the stable part of the coexistence equilibrium (see also Figure 2). For other combinations of *R*_max_ and *ε*, an ESS occurs in the coexistence region (dashed lines), but it is located in close proximity to the persistence boundary of the predator (black solid lines) if cannibalism levels are low. Consequently, any perturbation can induce a shift to the alternative stable CR‐equilibrium (indicated with blue shading) and thus lead to extinction of the predator. For higher levels of cannibalism, the ESS of *a*_jr_ is further away from the persistence boundary of the predator and lies in a parameter region with an unstable CR‐equilibrium. Text labels indicate the type of predator equilibrium with +PR = predator–resource equilibrium and +PCR = predator–consumer–resource equilibrium (coexistence state). All ESSs are convergent and evolutionary stable. Horizontal arrows indicate the direction of selection in the region of evolutionary suicide (red shading). Outside this region, direction of selection is always pointing toward the ESS. Values of all other parameters as in Figure 2

The model outcomes as presented in Figure 2 depend on maximum resource density (*R*_max_) and trade‐off shape (*ε*), but the implications remain qualitatively the same. For values other than *R*_max_ = 3 and *ε* ≤ 0, evolutionary suicide strictly speaking does not occur. Instead, there is an ESS for *a*_jr_ very close to the ecological threshold of predator persistence (solid black lines in Figure 3). Consequently, gradual evolution by small mutational steps will be stabilized just before evolutionary suicide occurs. However, any perturbation or large mutational step can push the predator population over the ecological threshold, which leads to predator extinction and a shift to the stable CR‐equilibrium. For *R*_max_ = 3 and *ε* = 2, the CR‐equilibrium is stable over the whole range of *a*_jr_ and predator invasion is impossible irrespective of the value of *a*_jr_. At high levels of cannibalism, the ESS for *a*_jr_ is close to the predator extinction boundary that is located at high *a*_jr_‐values (Figure 3). However, any perturbation will not lead to evolutionary suicide here, because the CR‐equilibrium overlapping with the ESS is unstable in this region.

### 3.2 Diet broadening of intraguild predators

**Figure 4:**
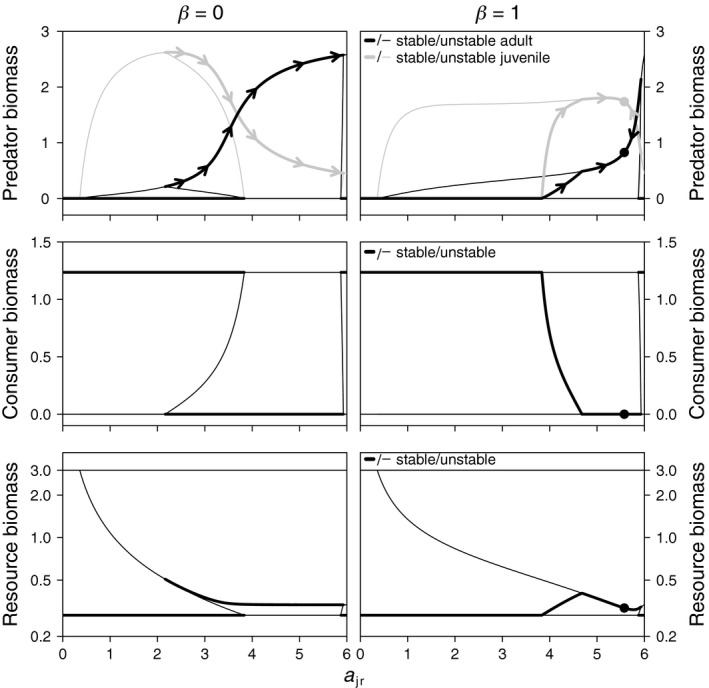
Model equilibria as a function of juvenile resource specialization (*a*_jr_; with concomitant change in *a*_ac_ following Equation (1)) for the diet broadening scenario with *a*_ar_ = 3 (adult predators feed on the resource, but consumers are superior resource competitors, irrespective of *a*_jr_). Evolutionary change in *a*_jr_ (indicated with arrowheads in top panels) leads to evolutionary suicide of noncannibalistic predators (*β* = 0; left panels), because the predator evolves toward high *a*_jr_‐values. This makes the PR‐equilibrium unstable and susceptible to consumer invasion. In case consumers invade, they outcompete predators and induce a shift toward a stable CR‐equilibrium. In case of cannibalistic predators (*β* = 1; right panels), there is a convergence and evolutionary stable ESS at high *a*_jr_‐values, where the PR‐equilibrium remains stable (resistant to consumer invasion). All other parameters as in Figure 2

For *a*_ar_ = 3, predators can persist solely on the resource (except for very low *a*_jr_‐values), but are inferior resource competitors compared to consumers (see Appendix). In this diet broadening scenario without cannibalism (*β* = 0), there is no stable coexistence of predators and consumers as a function of *a*_jr_ (Figure 4; Toscano et al., 2017). Predators persist in a stable PR‐equilibrium for intermediate to high *a*_jr_‐values, where adult density is sufficient to ward off invasion of competitively superior consumers. At lower *a*_jr_‐values, predator biomass distribution is dominated by juveniles and the low density of adults allows consumers to invade and outcompete the predator. This renders the PR‐equilibrium unstable, with the CR‐equilibrium being the only stable state in this region. At intermediate *a*_jr_‐values, this stable CR‐equilibrium co‐occurs next to the stable PR‐equilibrium. At *a*_jr_ ≈ 6, the predation attack rate *a*_ac_ reaches zero and consumers and intraguild predators only interact through resource competition. Since consumers are superior competitors (Appendix), the CR‐equilibrium is the only stable state here.

With a diet broadening, evolutionary suicide of the noncannibalistic predator occurs through a switch of the community attractor driven by selection toward juvenile as opposed to adult resource specialization (high *a*_jr_). Due to the lack of consumers in the PR‐equilibrium and the absence of cannibalism, predators derive no benefits from retaining a positive attack rate for predation. Consequently, selection is positive on *a*_jr_ and negative of *a*_ac_ (arrows along the PR‐equilibrium in Figure 4; top left panel). As such, predators become increasingly specialized as resource foragers. This eventually destabilizes the PR‐equilibrium if *a*_jr_ becomes close to its maximum value (*a*_p_ = 6). Here, consumers are released from predation by adult predators and are able to invade and outcompete the predator. Re‐invasion of the predator is prevented, because the CR‐equilibrium is the only stable state at high *a*_jr_‐values.

Also in the diet broadening scenario, does cannibalism disrupt the occurrence of alternative stable states and stabilize selection on the resource specialization trade‐off (Figure 4; right panels). With cannibalism, a stable coexistence state occurs in which selection on *a*_jr_ is positive (arrows along predator equilibrium in Figure 4; top right panel). Along with an increase in *a*_jr_, the PCR‐equilibrium changes into a PR‐equilibrium when consumers go extinct. In this PR‐equilibrium, there is a convergent and evolutionarily stable ESS for *a*_jr_ (indicated in Figure 4; top right panel). Because adult predators cannibalize juveniles, they benefit from retaining a positive predation attack rate and the predator therefore does not evolve toward *a*_jr_ = *a*_p_ = 6. The stabilizing selection induced by cannibalism prevents the evolutionary suicide of the intraguild predator.

**Figure 5:**
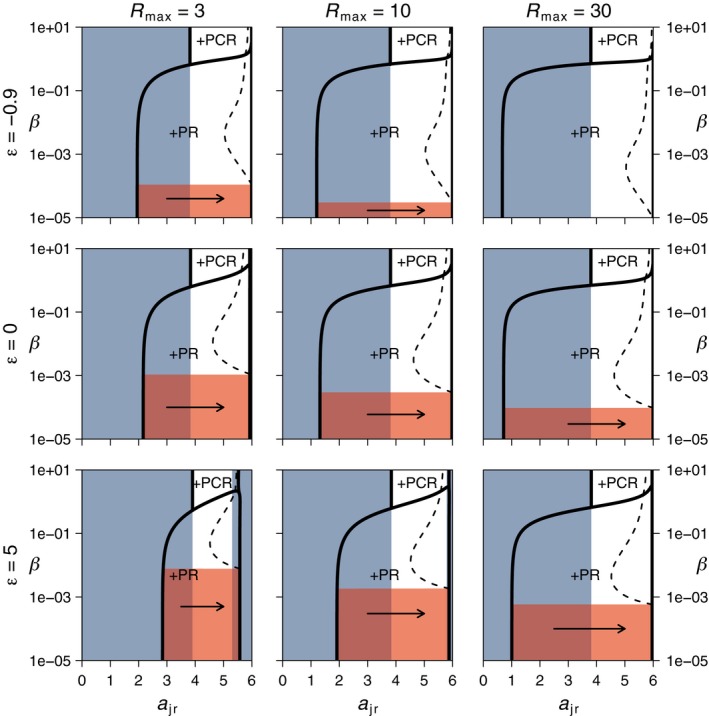
Increasing productivity and decreasing trade‐off strength decrease the regions of evolutionary suicide in the diet broadening scenario (*a*_ar_ = 3). Each panel shows the possible stable equilibria of the ecological dynamics as a function of *a*_jr_ and *β*, with *a*_ac_ related to *a*_jr_ following the trade‐off in Equation (1). The thick black lines are the parameter thresholds of predator persistence, with the text labels indicating the type of equilibrium that occurs (+PR = predator–resource equilibrium, +PCR = predator–consumer–resource equilibrium). Blue shading indicates a stable CR‐equilibrium that cannot be invaded by predators. Dashed lines show the evolutionary singular strategies (ESS) of *a*_jr_ that are convergent and evolutionary stable. In the red shaded region, there is no ESS for *a*_jr_ on a stable ecological equilibrium and selection on *a*_jr_ (indicated with arrows) leads to evolutionary suicide. Outside the red shaded region, direction of selection points toward the ESS. For high values of *β*, the predator persists in coexistence with the consumer and selection on *a*_jr_ is stabilizing. Values of other parameters as in Figure 2

Irrespective of maximum resource density (*R*_max_) and trade‐off shape (*ε*), the evolution of resource specialization in the diet broadening case is directed toward juvenile specialization for resource feeding and leads to evolutionary suicide if levels of cannibalism are low (Figure 5). Quantitatively, weak trade‐offs (low *ε*‐values) and high maximum resource densities (*R*_max_) decrease the threshold level of cannibalism (*β*) below which evolutionary suicide occurs and thus increases the region of cannibalism where selection on *a*_jr_ is stabilizing (red shadings in Figure 5). At high levels of cannibalism, predators persist in a PCR‐equilibrium and also here a stable evolutionary endpoint for *a*_jr_ occurs (Figure 5).

**Figure 6:**
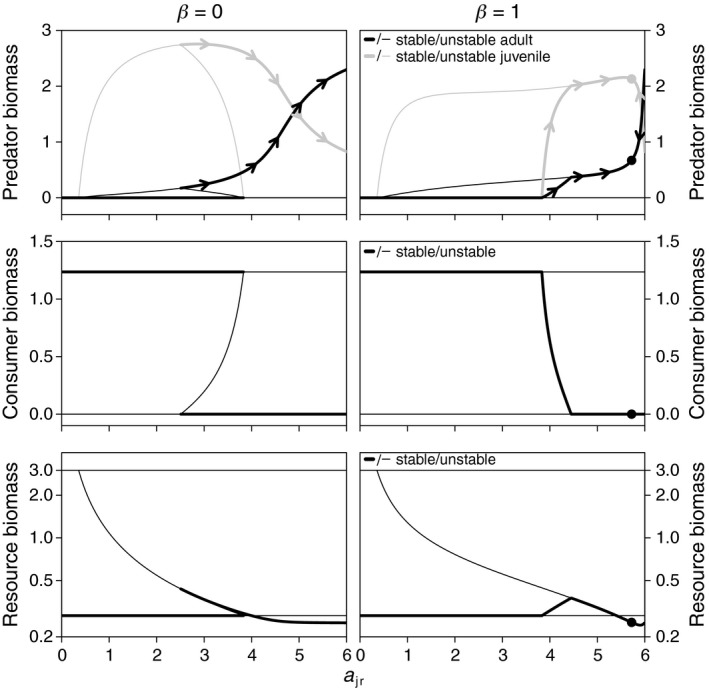
Model equilibria as a function of juvenile resource specialization *a*_jr_ for the diet broadening scenario with *a*_ar_ = 4 (adult predators feed on the resource and are superior competitors to consumers for *a*_jr_ > 4). For noncannibalistic predators, selection on *a*_jr_ (indicated with arrowheads in top panels) leads to the maximum value of juvenile resource specialization (*a*_jr_ = *a*_p_ = 6). In contrast to *a*_ar_ = 3 (Figure 4), this does not lead to evolutionary suicide, because the PR‐equilibrium remains stable at *a*_jr_ = 6. In case of cannibalism (right panels), selection is stabilized at high values of *a*_jr_. Values of all other parameters as in Figure 2

**Figure 7:**
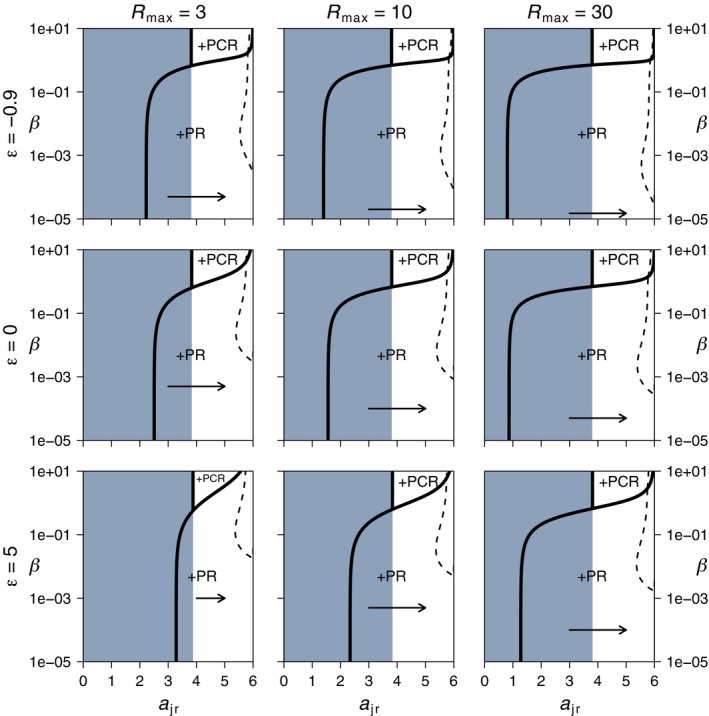
For *a*_ar_ = 4, intraguild predators can become superior in resource competition provided that *a*_jr_ > 4, in which case selection on the resource specialization trade‐off (Equation (1)) no longer leads to evolutionary suicide for low levels of cannibalism (*β*). There is still positive selection on *a*_jr_ (as indicated by the horizontal arrows), but predators persist in a stable PR‐equilibrium at *a*_jr_ = *a*_p_ = 6 and, following Equation (1), *a*_ac_ = 0. Higher levels of cannibalism lead to stabilizing selection on *a*_jr_, as indicated by the dashed lines that show the evolutionary equilibria (ESS) of *a*_jr_. All ESSs are convergence and evolutionary stable. Even higher levels of cannibalism lead to predators persisting in coexistence with consumers. All lines, symbols, and other parameters as in Figure 5

For *a*_ar_ = 4, the predator also undergoes a diet broadening, but becomes superior in resource competition for *a*_jr_ > 4 (Appendix). In contrast to *a*_ar_ = 3, the PR‐equilibrium cannot be invaded by consumers at high *a*_jr_‐values (Figure 6). As a consequence, positive selection on *a*_jr_ does not lead to evolutionary suicide in the noncannibalistic case and the resource specialization of juveniles becomes constrained by the value of *a*_p_, while *a*_ac_ evolves to zero (Figure 6; left panels). Consequently, the intraguild predator becomes a resource specialist and is no longer an (ontogenetic) omnivore. With cannibalism, there is again stabilizing selection in the PR‐equilibrium (Figure 6; right panels), leading to a convergent and evolutionary stable ESS at high resource specialization of juveniles. These results are qualitatively independent of the trade‐off shape (*ε*) and maximum resource density (*R*_max_; Figure 7). Similar to *a*_ar_ = 3, even higher levels of cannibalism lead to a stable PCR‐equilibrium that contains an ESS at high *a*_jr_‐values.

## 4 DISCUSSION

We study the evolutionary and ecological dynamics of a potentially cannibalistic, ontogenetic omnivore in a life‐history intraguild predation system, in which the ontogenetic omnivore (or intraguild predator) competes as a juvenile with its future prey (the intermediate consumer) (Pimm & Rice, 1987). Confronted with a trade‐off between specializing on early‐ vs. late‐life resources, ontogenetic omnivores can only stably persist over evolutionary time if adults cannibalize juveniles. Without cannibalism, directional selection increases resource specialization of one life stage at the cost of poor performance in the other life stage. If the intraguild predator cannot gain the competitive advantage over its prey (that is, if *a*_ar_ = 0 or *a*_ar_ = 3), directional selection compromises ecological persistence of the predator and can lead to evolutionary suicide. Alternatively, if the resource feeding rate of adult predators is sufficiently large (*a*_ar_ ≥ 4), directional selection leads to a competitive advantage over the consumer. Consequently, the noncannibalistic intraguild predator becomes a resource specialist and no longer persists as an (ontogenetic) omnivore. Cannibalism stabilizes selection on the resource specialization trade‐off and leads to a stable endpoint of evolution that is located away from the persistence boundary of the predator, or for higher levels of cannibalism, does not overlap with a stable consumer equilibrium in which predators cannot invade. As such, cannibalism prevents evolutionary suicide. Cannibalism leads to stabilizing selection because adult predators retain a predatory morphology (in the diet broadening scenario; *a*_ar_ = 3 and *a*_ar_ = 4) or because cannibalism changes the population regulation of the predator such that resource specialization of juveniles becomes important (in the diet shift scenario; *a*_ar_ = 0).

Although many species undergo ontogenetic diet changes as a result of ontogenetic growth (Persson, 1988; Pimm & Rice, 1987; Werner, 1988; Werner & Gilliam, 1984; Wilbur, 1980), there are relatively few empirical examples of ontogenetic trade‐offs in resource specialization. In Eurasian perch (*Perca fluviatilis*), an ontogenetic trade‐off has been shown to occur between the benthic and piscivorous life stages, which favor different body forms and feeding apparatus (Hjelm et al., 2000; Svanbäck & Eklöv, 2002, 2003). The costs of resource specialization are exemplified by the population of kokanee salmon (*Oncorhynchus nerka*) from Jo‐Jo Lake, Alaska. Ancestrally, these animals are adapted to benthic feeding, but have recently developed an ontogenetic diet shift to piscivorous feeding. Their maladaptiveness for the later prey type, however, leads to gill raker damage of up to 70% (Shedd et al., 2015). In addition, within species trade‐offs occur between morphs from different niches, such as benthic and limnetic forms of many freshwater fish species (Robinson & Wilson, 1996; Robinson et al., 1996; Schluter, 1995). Given the developmental coupling between different life stages (Cheverud, Rutledge, & Atchley, 1983; Marshall & Morgan, 2011; Moran, 1994; Werner, 1988) and the well‐studied phenomenon that different resources require different morphologies (Futuyma & Moreno, 1988), ontogenetic trade‐offs are likely to be important in more ontogenetic omnivores that lack a metamorphosis. More empirical work is needed to fully assess the prevalence of ontogenetic trade‐offs and its importance for the evolution of resource specialization. For example, experiments could compare the feeding ability on different resources between different life stages, or between different ecotypes that are thought to be at opposite ends of the resource specialization spectrum.

It well recognized that outcomes of evolutionary models depend critically on the nature and shape of the assumed trade‐off (Kisdi, 2006; de Mazancourt & Dieckmann, 2004). The ontogenetic trade‐off used here only involves the resource feeding rate by juvenile intraguild predators (*a*_jr_) and the predation attack rate of adult intraguild predators (*a*_ac_; Figure 1). Another biological feasible option would be to incorporate the resource feeding rate by adult intraguild predators (*a*_ar_) into this trade‐off, by coupling *a*_ar_ to *a*_jr_. The result of such a trade‐off would be a competitive system in which the intraguild predator specializes on resource feeding and outcompetes the consumer by evolving toward high values of *a*_jr_ and *a*_ar_. Qualitatively, this outcome is equal to our studied scenario of *a*_ar_ = 4, in which the intraguild predator evolves to become a resource specialist. To get a more complete understanding of the ecological and evolutionary persistence of ontogenetic omnivores, we have chosen not to incorporate *a*_ar_ into the ontogenetic trade‐off, and additionally study the scenarios in which the intraguild predator is prevented from evolving toward a resource specialist (*a*_ar_ = 0 and *a*_ar_ = 3).

### 4.1 Persistence of intraguild predators

An ontogenetic trade‐off in feeding efficiency was originally proposed as the reason for why intraguild predators are inferior in resource competition compared to their specialist prey (Persson, 1988; Werner & Gilliam, 1984). Moreover, the competitive superiority of prey was put forward as one of the requirements that enabled coexistence between intraguild predators and prey in the absence of size‐specific interactions (Holt & Polis, 1997; Mylius, Klumpers, de Roos, & Persson, 2001). The assumption of competitive dominance of prey is likely to hold in many (Byström et al., 2013), but certainly not all systems (Vance‐Chalcraft, Rosenheim, Vonesh, Osenberg, & Sih, 2007). The incorporation of size‐ or stage‐specific interactions has complicated the requirements for persistence of the intraguild predation module (in this case also referred to as life‐history omnivory/intraguild predation; Van de Wolfshaar et al., 2006; Hin et al., 2011; Toscano et al., 2017). Besides the competitive hierarchy between intraguild predators and their prey, coexistence in LHIGP systems is also determined by the presence and nature of an ontogenetic diet shift and the level of cannibalism. We present an overview of these theoretical predictions regarding ecological and evolutionary dynamics of LHIGP systems in Table 2. This table denotes the opportunity for persistence and coexistence by denoting both the stable population dynamical attractor and the evolutionary dynamics that result from selection on the ontogenetic trade‐off in resource specialization. Table 2 distinguishes between (a) the extent of diet change during the ontogeny of the intraguild predator, (b) three cases of competitive hierarchy between consumers and predators (see Appendix), and (c) the level of cannibalism. Considering both ecological and evolutionary processes, LHIGP systems are predicted to persist if levels of cannibalism (compared to interspecific predation) are not low and if intraguild predators do not suffer from competition in the juvenile life stage (Table 2).

**Table 2:** Overview of ecological and evolutionary dynamics of life‐history intraguild predation systems

			Competitive hierarchy		
			Consumers > all predators	Juvenile predators > consumers	All predators > consumers
*Diet broadening*					
Level of cannibalism	No/low	Ecological	ASS: PR/CR^a^	PR	PR^a^
		Evolutionary	Suicide	Suicide	Persistence^c^
	Medium	Ecological	ASS: PC/CR or only CR^a^^,^ d	PR	PR^a^
		Evolutionary	*Evolutionary transient to juvenile predators &gt; consumers*	Persistence	Persistence
	High	Ecological	Only CR^a^	PCR	PCR^a^
		Evolutionary	No persistence	Persistence	Persistence
*Diet shift*					
Level of cannibalism	No/low	Ecological	ASS: PCR/CR^a^^,^ b	PCR^a^^,^ b	
		Evolutionary	Suicide	Suicide	
	Medium	Ecological	ASS: PCR/CR or only CR^a^^,^ e	PCR^a^	
		Evolutionary	Persistence	*Evolutionary transient to consumers &gt; all predators*	
	High	Ecological	Only CR^a^	PCR^a^	
		Evolutionary	No persistence	Persistence	

Notes

Theoretical predictions of persistence of (cannibalistic) ontogenetic omnivores and coexistence with specialist consumers in life‐history intraguild predation systems. The table distinguishes three types of competitive hierarchy: (a) consumers are superior to predators, (b) consumers are superior to predators, but juvenile predators can grow in CR‐equilibrium, and (c) predators are superior to consumers; three levels of cannibalism: (a) no or low cannibalism, (b) medium cannibalism (lower or comparable to interspecific predation), and (c) high cannibalism (higher than interspecific predation); and whether the intraguild predator undergoes a diet broadening or diet shift over ontogeny (top vs. bottom part of the table, respectively). In case of a diet shift, predators cannot be competitively superior to consumers since adult predators do not feed on the resource. Possible ecological equilibria and dominant evolutionary process are indicated for each combination of competitive hierarchy, cannibalism level, and ontogenetic diet change. Evolutionary process represents either an increase in resource specialization of juveniles or increasing adult adaptation to predation as studied with the trade‐off in Equation (1).

ASS: alternative stable states; CR: consumer–resource equilibrium; PCR: predator–consumer–resource equilibrium; PR: predator–resource equilibrium.

a Toscano et al. (2017).

b Hin et al. (2011).

c Evolution toward maximum juvenile specialization and loss of ontogenetic omnivorous life history.

d Changes from PR/CR to only CR with increasing cannibalism.

e Changes from PCR/CR to only CR with increasing cannibalism.

The occurrence of cannibalism and the competitive hierarchy between juvenile intraguild predators and consumers in LHIGP systems were reviewed for a number of freshwater fish species by Byström et al. (2013). They concluded that large ontogenetic omnivores preferentially select conspecifics over interspecific prey (high values of *β* in our study) and that consumer species are more efficient zooplankton foragers than juvenile ontogenetic omnivores. The latter conclusion was based on attack rate measurements from separate feeding experiments (Byström et al., 2013). The high cannibalistic preference as observed by Byström et al. (2013) is in accordance with the requirements for predator persistence in LHIGP system that we pose here. However, it is difficult to draw conclusions about competitive inferiority of juvenile ontogenetic omnivores relative to their prey species based on higher attack rates alone, as other processes also contribute to competitive ability. These include (among others) handling time, maintenance metabolism, and conversion efficiency. The crucial experimental test would be to study whether juvenile ontogenetic omnivores can successfully grow and mature under resource conditions as set by the specialist consumer species. Furthermore, besides competition, other interspecific interactions such as interference or predation can also play a role in determining to what extent juvenile ontogenetic omnivores suffer from competition with specialist consumers under natural conditions.

### 4.2 Implications for the occurrence of cannibalism in nature

Cannibalism is a common interaction in terrestrial and aquatic food webs (Fox, 1975; Polis, 1981; Smith & Reay, 1991), especially for systems with substantial body size growth, such as the LHIGP systems studied here (Byström et al., 2013). Our results provide an explanation for the common occurrence of cannibalism in LHIGP systems, since we show that noncannibalistic LHIGP systems do not persist on evolutionary timescales. Furthermore, we find that low levels of cannibalism (relative to interspecific predation) can prevent evolutionary suicide (Figures 3 and 5). This result suggests that seemingly noncannibalistic systems could still be persisting because of the stabilizing selection offered by a small amount of cannibalism. This would imply that small levels of cannibalism lead to negligible changes in population dynamics, while having a large qualitative effect on the evolutionary outcome. Species with a diet shift in which cannibalism is absent can provide interesting insights about the applicability of our model. For example, a diet shift may result from an ontogenetic habitat shift that is induced by risk of cannibalism of adult/large individuals on juvenile/small conspecifics (Byström, Persson, Wahlström, & Westman, 2003; Foster, Garcia, & Town, 1988; Keren‐Rotem, Bouskila, & Geffen, 2006; Polis, 1981). In such case, a model structure with two independent resources that resemble the different habitats is more appropriate.

### 4.3 Evolution of resource specialization over ontogeny

Research on the evolution of resource specialization has mainly focused at the interspecific level, studying whether species evolve to become specialists or generalists (Futuyma & Moreno, 1988; Levins, 1962; Nurmi & Parvinen, 2008). A central result is that under a weak trade‐off (or a convex fitness set), generalists should evolve, while under a strong trade‐off (or concave fitness set), specialists should evolve (Levins, 1962; Ma & Levin, 2006). However, frequency and density dependence complicate this picture (de Mazancourt & Dieckmann, 2004; Rueffler, Van Dooren, & Metz, 2006). Resource specialization across ontogeny is much less studied (but see Ebenman, 1992 and Ten Brink & de Roos, 2017 for a theoretical treatment and Schluter, Price, & Rowe, 1991; Hjelm et al., 2000, 2003; German, Gawlicka, & Horn, 2014 for empirical work). Ebenman (1992) finds that for strong trade‐offs (corresponding to a large difference between juvenile and adult niche) selection favors juvenile specialization, at the expense of adult performance. For a weak trade‐off (small niche difference), specialization does not occur and an intermediate phenotype evolves. In the study of Ebenman (1992), adult specialization only occurs under a weak trade‐off and low productivity of the adult niche. Ten Brink and de Roos (2017) show that ontogenetic niche shifts evolve only if the adult habitat is sufficiently productive and juvenile performance in the original habitat is not hampered by the niche shift. Selection even favors maintaining high juvenile growth rates with adults being maladapted to their resource. In both Ebenman (1992) and Ten Brink and de Roos (2017), juvenile performance is more important than adult performance. Correspondingly, we find that most evolutionary stable outcomes are those with high resource specialization of juvenile intraguild predators (high *a*_jr_). Adult specialization is only observed in the diet shift scenario and always occurs in combination with a cultivation effect that nullifies competition in the juvenile stage (Figures 2 and 3). However, in all of these cases, there is an imminent risk of extinction for the predator, because the evolutionary equilibrium occurs close to the ecological persistence boundary and there exists an alternative stable CR‐equilibrium.

### 4.4 Evolutionary suicide in a community context

Evolutionary suicide occurs if a population adapts in a way that compromises its own persistence (Ferrière & Legendre, 2013; Parvinen, 2005, 2016). It is observed in a diversity of ecological models, but received only little attention from empirical workers (but see Fiegna & Velicer, 2003; Rankin & López‐Sepulcre, 2005). A common example of evolutionary suicide in population models is a population that evolves across a saddle‐node (or fold) bifurcation toward extinction, such as in the diet shift scenario studied here (Figure 2, see also Gyllenberg & Parvinen, 2001; Parvinen, 2005; Ferrière & Legendre, 2013). However, evolutionary suicide in the diet broadening scenario operates through a different mechanism. In this case, adaptation of the predator drives the system across a continuous transition in population dynamics, namely the invasion boundary of the consumer. Immigration of consumers then leads to an abrupt shift in ecological dynamics (attractor switching) and extinction of the predator. This possibility for evolutionary suicide occurs because we study evolutionary dynamics of the intraguild predator in a community context, therefore allowing for alternative community attractors (see also Patel & Schreiber, 2015). Since most studies on evolutionary suicide study species in isolation, or merely accompanied by a resource, we postulate that evolutionary suicide might be much more common than currently acknowledged if evolutionary dynamics are studied in a community context.

## 5 CONCLUSIONS

Size‐specific interactions prevail in natural communities, and many ontogenetic omnivores are likely involved in a mixed predation/competition interaction with a specialist consumer species. We extend the current body of theory on persistence and coexistence in these systems by studying evolutionary dynamics under an ontogenetic trade‐off in feeding ability between early and late resources. We show that evolutionary suicide limits persistence of a noncannibalistic intraguild predator, but cannibalism can lead to ecological and evolutionary stable persistence if juveniles can overcome the negative effects of competition with consumers. Our analysis shows that the requirements of species for persistence on ecological and evolutionary time scales differ and advocate for considering both processes simultaneously.

## CONFLICT OF INTEREST

None declared.

## AUTHOR CONTRIBUTIONS

VH and AMdR designed the research, VH analyzed the model and wrote first version of manuscript. VH and AMdR contributed to later versions of manuscript.

## Figures and Tables

**Figure A1:**
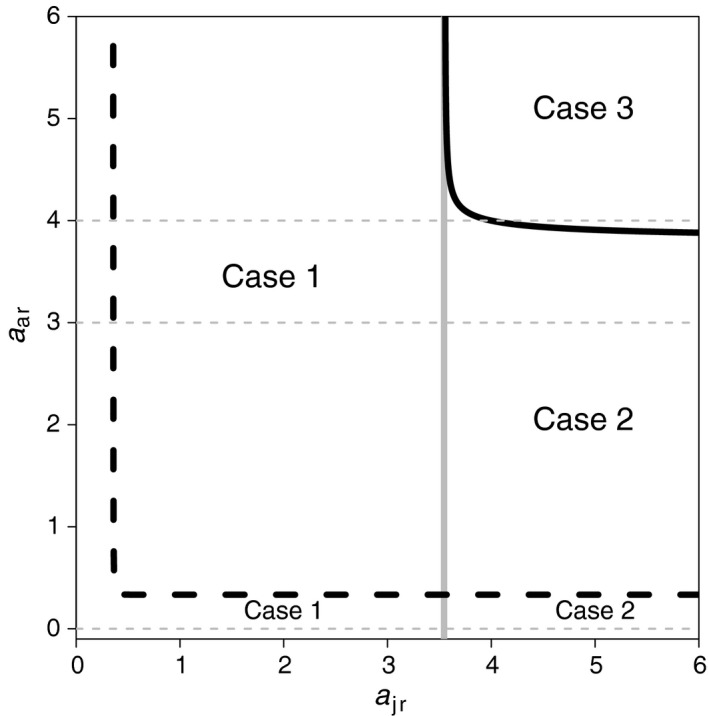
Different cases of competitive hierarchy between consumers and intraguild predators as a function of the adult attack rate on the resource (*a*_ar_; vertical axis) and the trade‐off in resource specialization (expressed as *a*_jr_ (horizontal axis), with concomitant change in *a*_ac_ following Equation (1), main text). With high specialization toward juvenile, resource foraging (high *a*_jr_ but low *a*_ac_) in combination with high resource feeding of adult predators leads to competitive superiority of predators (case 3). At lower *a*_ar_ (case 2), consumers outcompete predators, but juvenile predators are still able to grow with the resources in the CR‐equilibrium. The latter becomes impossible for *a*_jr_‐values below the gray solid line (in case 1; the gray solid line indicating $\nu_{j}\left(R_{c}^{*}\right)=0$). Below and left of the dashed black line, the predator cannot persist solely on the resource, which in terms of competitive hierarchy is the same as being outcompeted by the consumer. Horizontal dashed lines indicate the three different values of *a*_ar_ that are chosen for analysis

## Data Availability

There are no data to be archived. Code to produce the results and figures in this paper will be made available at https://bitbucket.org/vhin1/lhigpevo_pub/.

## APPENDIX 1 Competitive hierarchy between consumers and intraguild predators

### 1.1

Competitive hierarchy between consumers and intraguild predators has two aspects. Overall, competition between predators and consumers is determined in favor of the species with the lowest *R**‐value (e.g., the resource density that results in zero population growth [Tilman, 1980]). Beside the overall effect of competition, there can be stage‐specific competition between juvenile predators and consumers. In this case, consumers can prevent maturation of juvenile intraguild predators if the *R**‐value of consumers ($R_{c}^{*}$) is insufficient for juvenile somatic growth. Here, we outline how these two aspects of competition depend on the attack rate parameters of the predator (shown in Figure A1 in Appendix).

Overall competitive ability of the consumer and the predator are denoted by $R_{c}^{*}$ and $R_{p}^{*}$, respectively. The expression for $R_{c}^{*}$ follows from solving d*C/*d*t* for *R*, while setting *P*_a_ = 0, and is given by

(A1) $$R_{c}^{*}=\frac{T_{c}+\mu_{c}}{a_{\mathrm{cr}}\left(\sigma -h_{c}\left(T_{c}+\mu_{c}\right)\right)}$$

Next, we evaluate for which combinations of *a*_jr_ and *a*_ar_ the predator can persist in case $R=R_{c}^{*}$ combined with *C* = 0 and *β* = 0 and hence *D*_j_ = *μ*_p_. The condition for predator persistence is *R*_0_(*R*) = 1, where *R*_0_(*R*) is the expected lifetime reproduction of a single predator individual and given by De Roos and Persson (2013).

(A2) $$R_{0}\left(R\right)=\frac{\nu_{a}\left(R\right)}{\mu_{p}}z^{\mu_{p}/\nu_{j}\left(R\right)-1}$$

To assess the competitive ability of the predator in relation to the consumer, we evaluate $R_{0}\left(R_{c}^{*}\right)=1$. Evaluating this expression for the default parameters results in the solid black curve that is a function of *a*_jr_ and *a*_ar_ (Figure A1 in Appendix). To the upper right of this curve, predators outcompete consumers ($R_{p}^{*}<R_{c}^{*}$), while at the other side, consumers outcompete predators: ($R_{c}^{*}<R_{p}^{*}$). We furthermore assess the persistence boundary of the predator as a function of *a*_jr_ and *a*_ar_ by evaluating *R*_0_ (*R*_max_) = 1. This results in the black dashed curve in Figure A1 in Appendix. At the bottom left of this line, predators cannot persist solely on the resource.

Juvenile intraguild predators can grow with the resource density of the consumer‐resource equilibrium for $\nu_{j}\left(R_{c}^{*}\right)>0$. Evaluating this expression for the default parameters leads to the gray solid line in Figure A1 in Appendix.

Based on the different regions of competitive ability as shown in Figure A1 in Appendix, we choose three values of *a*_ar_ that, together with changes in *a*_jr_, cover all the qualitative competitive hierarchies between consumers and intraguild predators. Irrespective of *a*_ar_, juvenile predators can grow in the resource equilibrium as set by consumers for *a*_jr_ > 3.55. For *a*_ar_ = 0, adult intraguild predators do not feed on the resource and the overall superior competitive ability of the predator is not defined. This case corresponds to the diet shift scenario as studied by Toscano et al. (2017). For *a*_ar_ = 3, intraguild predators can persist on the resource but they are overall competitively inferior compared to consumers irrespective of the value of *a*_jr_. For *a*_ar_ = 4, intraguild predators can persist on the resource and are overall competitively superior to consumers for *a*_jr_ > 4.
